# Unsuccessful Stent Graft Repair of a Hepatic Artery Aneurysm Presenting with Haemobilia: Case Report and Comprehensive Literature Review

**DOI:** 10.1016/j.ejvsvf.2021.06.008

**Published:** 2021-06-26

**Authors:** Xing Gao, Jeroen de Jonge, Hence Verhagen, Wouter Dinkelaar, Sander ten Raa, Marie Josee van Rijn

**Affiliations:** a Department of Vascular Surgery, Erasmus Medical Centre, Rotterdam, The Netherlands; b Department of Hepatobiliary and Transplantation Surgery, Erasmus Medical Centre, Rotterdam, The Netherlands; c Department of Interventional Radiology, Erasmus Medical Centre, Rotterdam, The Netherlands

**Keywords:** Hepatic aneurysm, Haemobilia, Arterio-biliary fistula, Graft infection, Liver ischemia, Embolization

## Abstract

**Aims:**

To discuss treatment strategies for non-traumatic, non-iatrogenic hepatic artery aneurysms (HAAs) in the presence of an arteriobiliary fistula, illustrated by a case and followed by a comprehensive review of the literature.

**Methods:**

Following the PRISMA guidelines, 24 eligible HAA cases presenting with haemobilia were identified. Characteristics of patients, aneurysms, treatment strategies and their outcomes were collected.

**Results:**

A 69 year old patient with no previous hepatobiliary intervention or trauma, presented with jaundice and haemobilia caused by a HAA. Initial treatment by endovascular stenting was chosen to prevent ischaemic liver complications. Unfortunately, this strategy failed because of stent migration due to ongoing infection leading to a type 1A endoleak. The patient had to be converted to open surgery with ligation of the HAA. The patient recovered uneventfully and no complications occurred during the following 12 months.

**Comprehensive literature review:**

Of the 24 cases, nine had a true HAA and 15 were pseudo/mycotic aneurysms, mainly caused by endocarditis or cholecystitis. The majority were located in the right hepatic artery. In 20 cases, an endovascular first approach was chosen with embolisation, none with covered stents. Three of these cases had to be converted to open surgery because of rebleeding. In all open (primary or secondary) cases, ligation of the HAA was performed. One patient in these series died. No liver ischaemia or abscesses were reported, although one patient developed an ischaemic gallbladder.

**Conclusions:**

Patients who present with a HAA and haemobilia may be treated safely by embolisation or open ligation. Using a covered stent graft in these patients can cause problems due to ongoing infection and should be monitored closely by imaging. Publication bias and lack of long term follow up imply cautious interpretation of these findings.

## Highlights

In patients presenting with haemobilia in the presence of a non-traumatic and non-iatrogenic hepatic artery aneurysm

• Endocarditis and cholecystitis are the most common causes
• The main treatment modality is embolisation
• Liver ischaemia and liver abscesses have not been reported after treatment
• Close surveillance is recommended as the area has to be considered contaminated
• Treatment should be performed by a multidisciplinary team

## Introduction

Hepatic artery aneurysms (HAAs) are the second most common visceral artery aneurysms (VAAs) and the most common visceral pseudo-aneurysms. Due to its close relationship with the biliary ducts, rupture into the biliary tree is more common than into the intraperitoneal cavity.^1^ Regarding the complex hepatobiliary anatomy, surgical repair in this region is challenging and may lead to uncontrollable bleeding. An endovascular first approach is often chosen as a safer alternative or to serve as a bridge to surgery.^2^ However, embolisation by occluding the inflow and outflow of the HAA means interrupting arterial blood flow to the liver, which can lead to liver abscess, biliary necrosis and acute or chronic liver failure. Alternatively, the use of a covered stent will preserve blood flow, but when placed in a contaminated area, may maintain ongoing infection.^2^

In this paper, a case is presented with a true HAA complicated by haemobilia, initially managed by endovascular stenting to maintain the hepatic arterial circulation.^3^ Unfortunately, this approach was unsuccessful and the case was converted to open ligation. A comprehensive review of the literature of cases with haemobilia caused by non-traumatic, non-iatrogenic HAAs is also presented, focusing on the patient and HAA characteristics, HAA aetiology, treatment strategies and their outcomes.

## Case report

**Figure 1:**
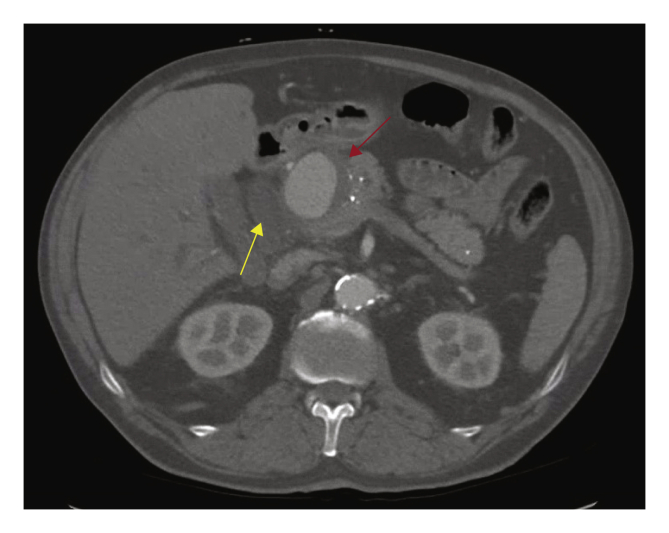
Computed tomography scan before intervention. Yellow arrow: dilated common bile duct, red arrow: true aneurysm of the common hepatic artery.

A 69 year old man presented with diarrhoea, weight loss, night sweats and progressive jaundice. Laboratory results showed a total bilirubin level of 413 μmol/L, a C reactive protein (CRP) level of 42 mg/L and a leucocyte count of 7.7 × 10^9^/L. A computed tomography angiogram (CTA) showed a HAA of the common hepatic artery (CHA) of 53 mm at its bifurcation with the gastroduodenal artery (GDA), compressing and dilating the common bile duct (CBD) (Fig. 1). The left hepatic artery (LHA) originated from the left gastric artery. Through endoscopic retrograde cholangiopancreatography (ERCP), a papillotomy was performed with placement of a plastic endoprosthesis in the CBD. Antibiotics were started and the patient was transferred to the academic hospital.

**Figure 2:**
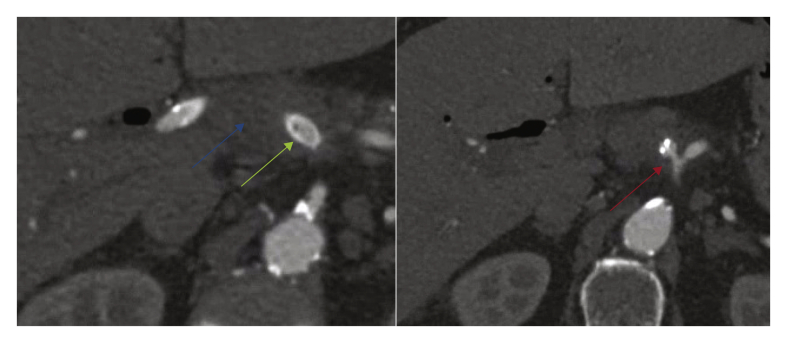
Day 1 post-endovascular intervention using a covered stent. Blue arrow: no flow in the hepatic artery aneurysm (HAA); green arrow: stent in HAA; red arrow: common hepatic artery origin.

Because of dysfunction of the endoprosthesis, replacement with an 8 cm covered self expandable metal stent (SEMS) during a second ERCP was performed. In the next two days, bilirubin and infection parameters increased, combined with a drop in haemoglobin levels. During a third ERCP, blood clots were seen and removed from the CBD, indicating the presence of an arteriobiliary fistula. A CTA was performed immediately showing signs of pending rupture of the HAA. To maintain the hepatic arterial circulation, 2 covered stents (8 × 50 mm Viabahn) were placed, using a percutaneous femoral approach, after the GDA was coiled. A subtraction angiogram showed an excluded HAA with patent flow through the stents and the right hepatic artery (RHA) (Fig. 2). Because of the open connection between the stents and the bile duct, antibiotics were continued.

**Figure 3:**
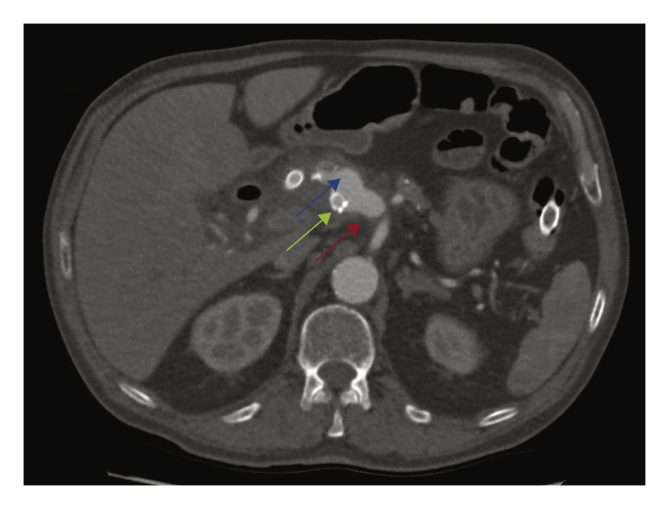
Day 19 post-endovascular intervention. Blue arrow: flow in hepatic artery aneurysm (HAA); green arrow: stent in HAA; red arrow: common hepatic artery origin dilated.

Three months later, the patient was readmitted with fever, increased infection parameters and positive blood cultures for *Pseudomonas aeruginosa*. A new CTA showed aerobilia without dilatation of intrahepatic bile ducts, an in stent thrombosis with limited flow and an excluded HAA. Antibiotic management was adapted but the patient was soon readmitted, because of a recurrent fever. A new CTA showed migration of the SEMS towards the transverse colon, and the origin of the CHA had dilated enormously creating a type 1A endoleak (EL). The aneurysm itself had grown in diameter with signs of local infection (Fig. 3).

**Figure 4:**
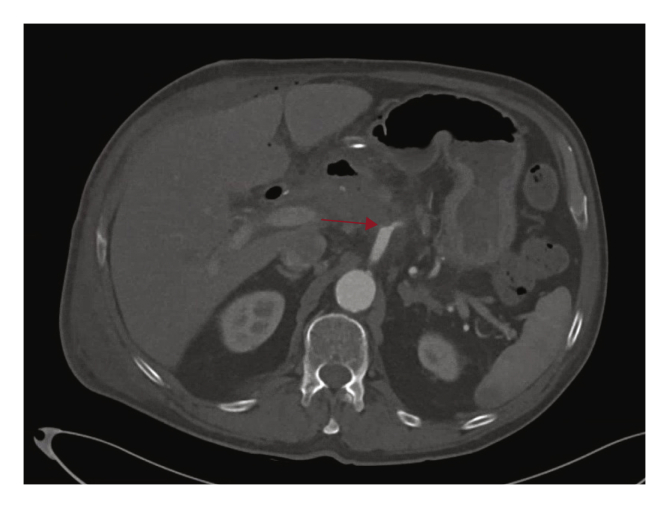
Five days post open repair by ligating the inflow and outflow artery of the hepatic artery aneurysm. Red arrow: common hepatic artery origin ligated.

After multidisciplinary consultation, open ligation of the HAA was considered to be the only permanent solution. Special consideration was given to the fact that ligating the CHA could result in ischaemia of the right side of the liver with risk of liver abscess formation. However, reconstruction of the artery in an infected area in the presence of possible continuous bile leakage, was considered too high risk. The operation was performed successfully by a team of vascular and hepatobiliary surgeons (Fig. 4). The post-operative course was uneventful. Liver enzymes on day one and 10 post-operatively were respectively; aspartate aminotransferase: 69→16 U/L, alanine aminotransferase: 155→22 U/L, gamma GT: 846→239 U/L, alkaline phosphatase: 600→141 U/L and bilirubin: 7→6 μmol/L. During his last follow up, 12 months after surgery, the patient had no signs of complications and the CTA showed no abnormalities. Written informed consent was obtained from the patient for publication of this case report.

## Comprehensive literature review

### Rationale and objective

Current guidelines recommend an endovascular first approach with emphasis on maintaining arterial flow to the liver to prevent ischaemic complications.^3^ However, this strategy, using a covered stent, failed in this patient and secondary open ligation of the HAA did not result in liver ischaemia or abscesses. A comprehensive review of cases presenting with haemobilia in the presence of a non-iatrogenic, non-traumatic HAA was performed, comparing treatment outcomes, complications and re-interventions.

## Methods

**Figure 5:**
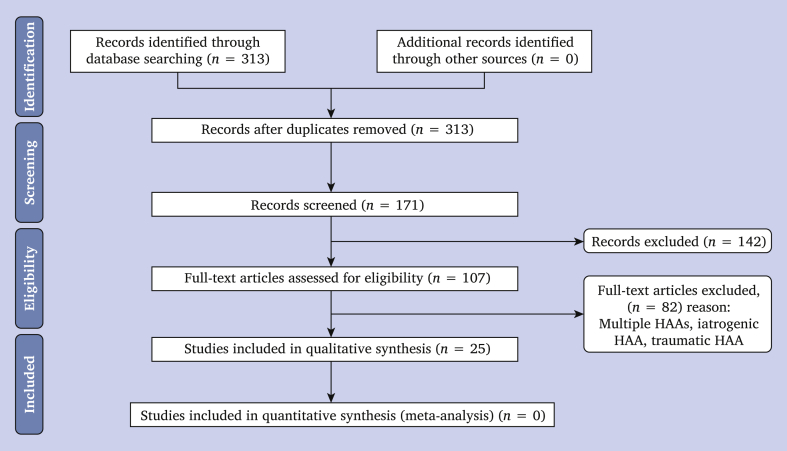
Preferred Reporting Items for Systematic Reviews and Meta-analyses (Prisma) flow diagram.

Two authors (X.G., M.R.) performed the search in November 2020 following the PRISMA guidelines.^4^ Studies were selected by searching the MEDLINE database. The following search quotes were used: (“Haemobilia “[Mesh] OR “arterio-biliary fistula” AND "Hepatic aneurysm”). Since endovascular approaches did not exist before 1994, only studies from then on were selected in order to take the choice of treatment into consideration.^5^ Only papers written in English were included. First, studies were screened by title and abstract. Second, all potentially relevant studies were selected (107 articles) according to availability and eligibility criteria using the full text article (Fig. 5). For the qualitative synthesis, studies of patients with multiple (>2) aneurysms and HAAs resulting from recent surgery, trauma, or endovascular intervention in the hepatobiliary tract were excluded. Continuous variables are presented as mean and categorical as count and percentage.

## Results

Twenty-four cases with a non-traumatic, non-iatrogenic HAA presenting with haemobilia were identified. Table 1 presents patient details as well as aetiology of the HAA, types of treatment and their outcomes. Nine cases had a true HAA and 15 were pseudo/mycotic. Age at presentation ranged from 5 to 83 years and female to male ratio was 1:1. The size of the aneurysm ranged from 1 to 4 cm. The majority was located in the RHA. Biliary obstruction was present in 12 cases (50%) at time of presentation. Endocarditis and cholecystitis were the most common causes of the pseudo/mycotic HAAs. In all but 4 cases, an endovascular first approach was chosen with embolisation of the HAA. No patients were treated with covered stents. One case was treated only by removing blood clots through ERCP and the HAA disappeared on follow up imaging.^19^

**Table 1:** Patient and aneurysm characteristics, types of (re-)intervention and complications

Year and author	Age – y	Sex	Etiology of HAA	HAA diameter – cm	Biliary obstruction	Location	First treatment	Intervention	Complication	Time to re-intervention	Type of re-intervention	Follow up time
1997 Corr^6^	5	F	Ascariasis	unknown	No	Left HA	Endovascular	Embolisation: microcoil	None			2 weeks
2003 Ferrari^7^	24	M	Tuberculosis	Unknown	Yes	Unknown	Endovascular	Embolisation: coils, gelfoam, lipiodol	Re-bleeding	1 week	Open ligation	1 day, patient died
2003 Liu^8^	74	F	Cholangitis	“Small”	No	Middle HA	Endovascular	Embolisation: coils	None			2 years
2003 Rai^9^	47	F	NHL/cholangitis	Unknown	Yes	Right HA	Endovascular	Embolisation: n.s.	None			14 months
2004 Akatsu^10^	64	F	Cholecystitis	3.0	No	Right HA	Endovascular	Embolisation: microcoils	None			5 years
2006 Hatzidakis^11^	40	M	Behçet's disease	Unknown	No	Right HA, from SMA	Endovascular	Embolisation: coils	None			2 months
2006 Traversa^12^	49	F	Unknown, degenerative	Unknown	No	Common HA	Endovascular	Embolisation: coils	None			5 months
2008 Chirica^13^	61	M	Atherosclerosis	4.0	Yes	Common HA	Surgical	Ligation	None			9 months
2009 Lin^14^	73	M	Cholecystitis	2.0	Yes	Right HA	Surgical/Endovascular	HAA discovered during cholecystectomy. Embolisation (n.s.) 2 days later	None			2 years
2010 Arroja^15^	72	M	Cholecystitis	Unknown	No	Right HA	Endovascular	Embolisation: n.s.	None			2 years
2010 Trakarnsanga^16^	55	M	Unknown, degenerative	4.0–5.0	Yes	Common HA	Endovascular	Embolisation: histoacryl	None			2 years
2011 Mortimer^17^	51	M	Endocarditis	2.0	No	Right HA, from SMA	Endovascular	Embolisation (coils) after negative emergency laparotomy because of haemodynamically unstable patient with unknown source of bleeding	None			None
2012 Bibyan^18^	64	M	Cholecystitis	“Large”	Yes	Right HA, from SMA	Endovascular	Embolisation: n.s.	Calculus of aneurysm blocking gallbladder	“few” days	Laparoscopic cholecystectomy	None
2012 Yu^19^	61	F	Pancreatitis	Unknown	Yes	Left HA	None	Removal of blood clots through ERCP, stable thrombus in pseudoaneurysm	None			3 months
2014 Komatsu^20^	53	M	Marfan syndrome	6.8	Yes	Unkown (adjacent to PV)	Endovascular	Embolisation: coils	haemobilia	9 and 11 days	2nd and 3rd embolisation left HA and laparotomy with open resection and left hemihepatectomy	3 years
2016 Vultaggio^21^	89	F	Atherosclerosis	1.0	No	Right HA	Endovascular	Embolisation: microcoils	Cholangitis	2 months	Antibiotics	None
2017 Bacalbasa^22^	68	n.a.	Unknown, degenerative	Unknown	No	Common HA, proper HA and GDA (Left HA originated from the left GA)	Surgical	Right PV embolisation for left liver hypertrophy followed by resection of the aneurysm without initially planned right hepatectomy	None			None
2017 Bacalbasa^23^	66	M	Unknown, degenerative	Unknown	Yes	Right HA	Endovascular	Embolisation: polyvinyl alcohol particles, gelaspone and detachable spirals	Pancreatitis and retrograde filling of HAA and PV rupture	3 weeks	Open resection HAA and segment of PV with roux and Y, complicated by abscess subhepatic which was drained	3 months
2018 Delgado^24^	65	M	Endocarditis	“Small”	No	Right HA	Endovascular	Embolisation: n.s.	None			None
2018 Fong^25^	34	F	Endocarditis	2.8	Yes	Right HA	Endovascular	Embolisation: coils and thrombin	None			None
2018 Warren^26^	53	F	Endocarditis	1.8	Yes	Right HA	Endovascular	Embolisation: coils	None			4 days
2019 Das^27^	72	F	Unknown	Unknown	Yes	Right HA	Endovascular	Embolisation: coils	None			None
2019 Zhu^28^	28	F	Endocarditis	30	No	Proper HA and left HA	Surgical	Ligation of both aneurysms and cholecystectomy, T tube drain of CBD	None			3 months
2019 Saeed^29^	83	M	Unknown, degenerative	“Large”	Yes	Common HA	Endovascular	Embolisation with coils (no haemobilia at that point)	Flow in aneurysm and haemobilia	5 months	Recoiling and cholecystectomy for ischaemic gallbladder	None

HAA = hepatic artery aneurysm; F = female; M = male; cm = centimetres; NHL = non-Hodgkin's lymphoma; HA = hepatic artery; SMA = superior mesenteric artery; GDA = gastroduodenal artery; PV = portal vein; GA = gastric artery; ERCP = endoscopic retrograde cholangiopancreatography; CBD = common bile duct; n.s. = not specified.

No complications were reported for the open cases. In an “open” case, the HAA was discovered during cholecystectomy.^14^ An accidental rupture was sutured and embolisation was planned 2 days later. Another open case described a large HAA originating from the common and proper HA and the GDA, with an aberrant LHA.^22^ Percutaneous right portal branch embolisation was performed to induce left hemiliver hypertrophy and two months later, the HAA was resected without reconstruction. A hemihepatectomy was not performed, because vascularisation of the liver remained patent through the LHA. One uncomplicated open ligation of 2 HAAs (proper and LHA) was performed after a failed attempt to embolise the HAAs (the aneurysms could not be reached). The patient had an almost complete occlusion of the CHA, and the RHA originated from the superior mesenteric artery.^28^ Blood was supplied to the proper HA through the left gastric artery.

Of the 20 embolisations, 6 (30%) had a complicated outcome. Three of them (15%) were converted to open ligation. In the first case, rebleeding occurred after embolising the HAA. Massive blood loss resulted in death the day after surgery.^7^ The second case was converted because of recurrent haemobilia.^20^ During laparotomy, adhesion of the aneurysm to the liver was too dense to be dissected, requiring a left hemihepatectomy. In the third case, a choledochal–aneurysmal fistula and portal vein (PV) rupture were found during laparotomy.^23^ The HAA was resected *en bloc* with segmental resection and reconstruction of the PV and CBD. The other 3 complications consisted of cholangitis and an ischaemic gallbladder for which antibiotics were given combined with a cholecystectomy in 2 patients.^18^^,^^29^ Of the 24 patients, 13 were followed up after discharge (3 months to 5 years). No liver abscesses or liver ischaemia were reported.

## Discussion

HAAs are the second most common VAAs and most common visceral pseudo-aneurysms. Mycotic, symptomatic and pseudo-aneurysms, should always be treated regardless of size. An endovascular first approach with emphasis on maintaining arterial flow to the liver was recommended in the recently published Society for Vascular Surgery guidelines on the management of visceral aneurysms.^3^ These guidelines recommend stenting, open reconstruction, or in case of coiling large intrahepatic HAAs, resection of the involved part of the liver to prevent necrosis. Maintaining vessel patency is certainly mandatory when the PV is occluded, or even stenosed, to prevent liver failure.^31^ There are no specific recommendations for HAAs presenting with haemobilia.

HAAs causing haemobilia are extremely rare when compared with other causes like percutaneous interventions, ERCP, or surgery.^30^ Prompt diagnosis is essential, but often overlooked in the absence of previous interventions in the hepatobiliary region. The classic triad of right upper quadrant pain, jaundice, and overt upper gastrointestinal bleeding (Quincke's triad) is only present in 25%–30% of the patients. Haemobilia can be diagnosed with upper endoscopy, ERCP, CTA, and/or endoscopic ultrasound. Simultaneously, the cause of haemobilia should be identified and treated. An HAA can be confirmed on CTA or angiography. Management is based on two main principles: haemostasis and maintaining bile flow.

Evidence based haemostatic treatment strategies for HAAs with haemobilia are hard to propose in the absence of large studies. A meta-analysis studied treatment of pseudo-aneurysms in 100 cases after laparoscopic cholecystectomy.^2^ The most common presentation was haemobilia (85.1%). The main treatment strategy was embolisation (72.3%), while stent grafts were used in only 4 patients. Ten patients (13.7%) developed liver abscesses and 9 (12.3%) hepatic ischaemia. In another study, 83% of patients developed ischaemic liver injury after hepatic artery embolisation for haemorrhage following hepatobiliary surgery.^31^ Last, Mezhir et al. showed that when liver tumours or haemorrhage are treated by hepatic artery embolisation, liver abscess formation was especially common in patients with a bilio-enteric anastomosis (33%) or an incompetent sphincter (10%).^32^ Based on these data, it was expected that embolisation of a HAA in the presence of a hepatobiliary fistula would also lead to liver ischaemia and abscesses. However, none of the 24 cases included in the review developed such a complication, even though no patients were treated by a blood flow preserving method. It is important to realise that long term follow up for most of these cases was missing and that transient liver ischaemia might have been missed or not reported. Publication bias might also have directed towards better outcomes.

Fifty per cent of the cases presented with biliary obstruction at time of haemobilia. It is hypothesised that if instrumentation of the biliary system has not yet been performed, percutaneous biliary stent placement without disruption of the papilla, may be useful to prevent bacterial contamination of the biliary tree, thereby reducing the risk of liver abscess formation after embolisation. In the endovascular group, 10 cases presented with jaundice of which 6 underwent an intervention (plastic stent, nasobiliary/internalised drainage or stone extraction). Bile leakage was not reported in any of the uncomplicated endovascular treated cases. Of the 3 cases that were converted to open surgery, all underwent biliary diversion around the time of embolisation, as did the present case. During open surgery, biliary repair was performed only in 1 patient. Of the 4 primary open cases, 2 had per-operative T tube insertion, and all ended uneventfully. Overall, in 70% of all open surgery cases, a biliary repair or diversion was performed at some point. A recommendation on bile duct strategy is difficult to propose based on these small numbers.

## Conclusion

Hepatic artery aneurysms presenting with haemobilia should be excluded promptly. These patients may be safely treated by embolisation or open ligation, as none of the 24 cases in this comprehensive review were treated with a blood flow preserving method and no ischaemic liver complications or abscesses were reported. Conversely, using a covered vascular stent in these patients can cause problems due to ongoing infection.

## Conflict of interest

None.

## Funding

None.
